# Ovarian Tumors

**Published:** 1862-01

**Authors:** Wm. H. Byford

**Affiliations:** Professor of Obstetrics and Diseases of Women and Children, in the Medical Department, Lind University, Chicago, Illinois


### CHICAGO MEDICAL EXAMINER

N. S. DAVIS, M. D., Editor.---FRANK W. REILLY, M. D., Ass't Editor.

VOL. III.] JANUARY, 1862. [NO. 1.

### Original Contributions.

ARTICLE I.

### OVARIAN TUMORS.

By Wm. H. BYFORD, A. M., M. D.,

PROFESSOR OF OBSTETRICS AND DISEASES OF WOMEN AND CHILDREN, IN THE MEDICAL DEPARTMENT, LIND UNIVERSITY, CHICAGO, ILLINOIS.

### II.—Anatomy, Nature and Pathology of Ovarian Tumors.

Ovarian tumors spring from, and are for the most part formed of the hypertrophied tissues of the ovaries. There are tumors very much resembling, and often mistaken for them, however, developed in the lateral ligaments. These latter are generally distended and hypertrophied sacs of the parovarium, and contain thin serum, merely; are cured sometimes by merely tapping, or spontaneously burst or are broken by accident in the peritoneal cavity, and disappear, never again to annoy the patient. The fallopian tubes sometimes are the seat of enlargement. The tubical canal becomes obliterated at each end, and fills up with the hilus usually appropriated to the lubrication of its inner surface, hypertrophies in tissue and thus constitutes a morbid development. Although rare, these two forms of tumors are observed. Doubtless, other enlargements of a nature not yet properly understood, are sometimes originated by unknown causes. The anatomical distinctions can be made in most instances by careful dissections, so that in cadaverous investigation, they need not be confounded with the ovarian tumors, except it be where all tissual distinctions are obliterated by an inter-current or supernatant disease. It is not my purpose, now, to pursue this subject further than the mere mention here made. In the proper ovarian tumors, we may trace three coats or layers of tissue forming their walls. The external is the serous or peritoneal. It is shining and smooth as this membrane is elsewhere, and seldom changed in anyway, except it may be thickened and hypertrophied. It can be traced into the peritoneal covering of the viscera and abdominal parietes, and consequently needs no elaborate description. The internal coat or lining membrane, is doubtless the membrana granulosa of the ovisac, very much hypertrophied. When small, something like epithelium seems to be its entire composition. As they grow and develop, the epithelial arrangement is less perfect, until when very large, we can observe it only in patches. In many cases when thus large, this membrane has a smooth, lustrous appearance, but in others, it is more or less thickly studded with granular projections, varying from almost imperceptible minuteness, to the size of peas, or even larger. Regarding the main sac as an hypertrophied ovisac, I think these little granular sacs (for they prove to be sacs upon examination), are also of the same nature, and the origin of the numerous endogenous or supplementary growths which constitute one of the polycystic varieties.

The middle coat is made up from the stroma of the ovary. Its strength depends upon quite a considerable amount of fibres, which enter into its composition. As the tumor develops, these fibres are enlarged, and apparently if not really, increase in numbers, until they constitute the most of the thickness of the walls, and in some parts, make quite a thickness, density and toughness of tissue. These qualities are greater in old large sacs than in the smaller and younger ones. At the pedicle and for some distance up the sides, they are greater than in other portions, being in these parts sometimes a quarter of an inch thick, while at the fundus or distal portion, they may be thin and fragile. The whole of this coat may be very tough and thick, so as to resist great force, or it may be thin throughout, so as to be easily ruptured at almost any point. Entangled in the meshes of these fibres may be discovered, in many cases, the minute microscopic points, so numerously scattered through the substance of the ovaria. These points are believed to be the origin of the germinal spot in the ovum by some physiologists, and around which are developed the ovum and progressively the whole ovisacs and their contents; and I believe, that their presence in the walls of the tumors over much, if not the whole of their extent, accounts for the development of the minute granular internal projections above described. In a tumor recently removed from the body, we may not unfrequently, by holding it up to the light, discover the peculiar buffy tinge seen in the stroma. The vessels are situated in this coat. They are numerous and some of them large, so large, that great care is necessary to prevent them from bleeding when the peduncle is divided. They are developed (it is hardly necessary to say), to this great size, from the minute twigs which penetrate the substance of the ovary.

The shape of ovarian tumors may vary much. They may be regularly globular, polyglobular, angular, or irregular in almost every way. When small, the ovary may be seen as constituting a considerable portion of the tumor. When large, the ovary may be almost lost in the walls, or observed as a mere tubercle sticking to, or imbedded in its side. Very generally, but one ovary is the seat of disease, but in a few instances, both are affected. Not often do they become consecutively the nidus of these growths; one being first the subject of disease, and then followed by the other. And when such is the case, we are not warranted in supposing the one to be the cause of the other, either remotely or directly. Notwithstanding the above assertion, I do not wish to be understood to say, that sympathetic degeneration between these two bodies is impossible. These tumors divide themselves anatomically into monocystic and polycystic. The one having a single cystic cavity, the other several. The polycystic variety is formed by the development of several cysts adjoining, or by the side of each other, and independently attached to, or springing from each other on the external surface, or within the cavity of one large one. The instances of polycysts growing by the side of each other, and being independently attached, resembles at first the single. At an early stage of development, they may stand free of contact—one with the other; but as they grow in size, in consequence of the small surface of the ovary to which they are attached, they crowd together, so that it is not always easy to say whether they were not developed from each other. The cysts from which smaller ones grow, are called proliferous. They are doubtless single for some time in their early development, but carrying up, as they increase in size, the proper substance of the ovary, with its rudimentary ovisacs, after a while the inner or outer surface is bulged by the maturity of these last, which, if they do not dehisce and allow the escape of the ovum, still grow into a subordinate tumor. This process is separate, until there is a glomeration of cysts to quite a number, from four to fifty, of various sizes,—from the size of a man's head, down to that of a pin's head. Small ones may be so numerous as to stud a large part of the inner surface with granulated elevations. This is the most frequent variety met with in practice.

There is a great difference in the sensible qualities of the contents of the cysts, in different cases, and of the different cysts in the same case. In some, it is very thin, in others, very thick, and tenacious, while the color shades from black, inky, to liquid clearness. The monocystic, as a general thing, affords thinner, clearer fluid than the polycystic, though this is not invariably so. It is in the monocystic variety, however, that we generally find the solid contents. These solid contents are, for the most part, formed of tegumentary, adipose, hairy, osseous and dental tissues. All these are irregularly developed and distributed, so that no semblance in shape or other conditions can be discovered to an independent being, —as a fœtus. Sometimes the tegumentary substance is small and gives attachments to a few hairs, sometimes the quantity of hair is long and entirely isolated, and either inextricably knotted together, or straight and few in number. They may be long,—ten or twelve inches, or very short. Irregular bones, or the enamel of teeth, without the bony structures, are often attached to the side of the cavity; but no organic order, symmetry, or completeness, has yet been seen. In fact, should a femur, a scapula, a complete maxilla or other complete organs be developed, the tumor would unquestionably range itself under the head of extra-uterine fœtation, and not ovarian disease proper. The tumor containing solid materials of this kind is usually small. Not unfrequently large fibroid growths are observed in the ovary at the base of a single or multiple cystic tumor. These solid fibroid or fibrous growths may be simple or benign in their nature, or malignant. This complication of ovarian dropsy, I think more frequent in persons advanced in years (over forty), than younger ones. The contained fluid of the polycystic tumor, is ordinarily highly albuminous, of high specific gravity, tenacious, and more or less colored. The fluid is so thick sometimes, as not to flow through a small canula. Blood and pus are the coloring matters of this fluid ordinarily. From one tumor of several cysts, I drew pus from one cyst, dark coffee-ground, sanguino-serous fluid; from another, a beautiful straw color; from still another, a fluid of a delicate azure tint. After tapping, more or less alteration is observed in the fluid, each operation withdrawing fluid affected by chemical or pathological circumstances. In the former putridity or acridity would result, in the latter, the purulent productions of inflammation might be expected, or fibrous concretion, or serum changing the tenacity and thickness of the fluid.

There are some chemical and microscopic resemblances in the fluid from almost all varieties of ovarian tumor. Albumen seems to be almost always present; in some specimens of fluid, strong acids, or heat, causes it to assume a solid form, coagulating and adhering like the white of an egg when cooked in boiling water; in others a small precipitate is all that is observed. Between these extremes, all shades of difference exist, but undoubtedly nearly all ovarian tumors yield highly albuminous fluid. The reaction is alkaline.

Mr. Nunn says that, "as the results of many examinations (microscopic) of different specimens of ovarian fluid, the most constant characteristic of such fluid, is its containing in greater or less abundance, cells gorged with granules; and in addition, circumambient granules having the same measurement encompassed by the cell. The size of the gorged cells and included granules varies greatly even in fluid from different cysts in the same ovary." This description of fluid could, with certainty, remain good of the first evacuation only, as pus and blood globules are not unfrequently found in subsequent evacuations.

I have already stated what I believe to be the nature of the sacs or cysts of ovarian tumors, and it will not be necessary to repeat it; but there is one curious question as to the origin of the solid substances sometimes found in them, which so very much resemble the tissues of a fœtus, upon which I feel at liberty to add my conjectures to those of many others, who have written upon ovarian disease. I do not desire to argue elaborately in favor of the opinion I embrace, or against those who adopt a different one, nor to give instances to any extent in proof for, or against. I do not think that the discovery of hair, fat, teguments, bone or teeth, in the ovary in the imperfect state of development, and irregular relationship above described, affords any evidence of inpregnation or sexual connection. In explanation of the formation of these tissues, it may be conjectured that the ovaria have such independent cell capacities as will enable them not to form a living being independent of sexual influence, but under certain circumstances, to imperfectly produce tissues of a very elementary nature, resembling those found in the products of impregnation. The tissues of ovarian disease are of the lower grade in a formative sense. We do not find muscle, nerve, or vessel in these places, and under these circumstances. There does not seem to be generic energy enough in the ovum, unaided by seminal impression, to give the direction to cell action for the formation of these more complicated tissues. We should observe, too, that although the cell action of the ovum may start up the formation of these singular tissues, there is no generic order or completeness in that action. In all this, we see another of nature's distinct modes of formation under peculiar circumstances. I am not aware that in any other part of the body, such a condition of development ever takes place inside of a cyst,—a development which gives origin to so irregular an assemblage of tissues, without the formation of any organ out of them. When fœtal tissues are found elsewhere in the body, or in any part of the male, there is something of organic completeness about them, and perfect bone, tooth, or some or many organs may be distinctly traced; nerve-matter, muscle, etc., and in fact, all the more complicated tissues are met with. These facts, I think, establish a broad difference between the products of conception and the irregular cell or tissual development of the ovum, and I cannot see why the theory I have mentioned, may not explain all the circumstances fully. The pathological condition of the system which gives origin to this affection, is not determined, and perhaps, is not peculiar; in fact, it is probable that the general condition of the patient has nothing to do with the production of ovarian tumors, particularly the dropsical variety. Dr. F. Bird thinks that it is found more frequently in scrofulous or strumous persons, and appears in families subject to phthisis. Statistics have not been resorted to in sufficient copiousness to prove or disprove this opinion. It, according to Dr. Bird, occurs more frequently in patients from twenty-five to thirty-five years of age; is more common in the married than single ladies, and is generally associated with sterility.

I have not much doubt, however, that the state of the ovaries has much to do in starting up these tumors, and it is most likely that inflammatory thickening or condensation of the peritoneal or fibrous envelops of this organ, is the condition. If we imagine the fibrous indusium to be thickened and condensed, so as to be less fragile or less amenable to the influence of the absorbents, and thus, failing to yield to the distension of the maturing ovum, so confine it that the follicular fluid cannot escape, we have incipient ovarian dropsy.

It is only necessary that the unruptured coats of the ovisac should continue to secrete its fluid, and the strong indusium refuse to crack open and allow its escape, to have all the conditions of an ovarian dropsy, that will be limited only by circumstances foreign to those of their origin. However this may be, there can be no question, but that the beginning is the failure to discharge the fluid of the ovisac, and that this last is, in the early stages, the fluid of the ovarian tumor. There is, also, a kind of independent pathology of ovarian tumors: After they are developed to a certain extent, they become subject to accidents and disease, and play an important part in consequence of this fact in the sanitary conditions of patients in whom they exist. Inflammation for instance, attacks them, and causes ulceration in their walls, so as even to perforate them, making a communication between the cavities of contiguous cysts, or with the peritoneal cavity. Without perforating the walls of the tumor, the ulceration may produce a good deal of pus, which is mingled with the other contents of the cyst in which it occurs. General inflammation of its walls may proceed to a fatally exhaustive extent, or spread to the peritoneum, and thus indirectly cause death. Gangrene may also result, which may be confined to the cavity of some of the cysts, and induce a putrid, offensive state of the contents, or perforate the partitions dividing, and thus make a communication between cysts, or open them into the peritoneal cavity. The walls may also rupture from distension, in consequence of their becoming attenuated; or by a violent stroke or fall, or other shock, the tumor may be ruptured, and the contents escape into the peritoneal cavity. By means of ulcerative communication with the fallopian tubes, evacuation of the fluid occurs. Adhesion to the walls from inflammation and ulceration through the parts thus agglomerated, sometimes results, and the fluid so discharged. Inflammation also causes adhesion at various parts. The fibrin effused, glues it to the surrounding parts,—the abdominal walls, the intestinal canal, bladder, and other viscera. Slight inflammation is supposed to increase the effusion in their cavities, and cause them to grow very rapidly. Inflammation, also, sometimes, no doubt, causes obliteration of the cavity from adhesion of the walls. This is more frequently the case when it results from external causes,—as blows, tapping, pressure, injection, etc. Now it hardly ever happens that these diseased conditions and accidents of the tumors fail to produce their effects upon the health of the patient. No doubt, but that death occurs from extensive disease in the sac without any organ being involved in the trouble directly. A large production of pus would exhaust the patient ; gangrene to a large extent would cause death, as extensive gangrene of unimportant organs generally does. But an extension of disease to the peritoneum and surrounding viscera, or by the effusion of the acrid contents of a diseased cyst, are more likely to be the modes of progress to constitutional disturbances inaugurated by inflammation in the tumors. When the tumor is burst, and its contents effused into the peritoneal cavity, the peritoneum seldom escapes without inflammation ; but the degree will depend upon the nature of its contents. If they are not vitiated, but consist of the bland albuminous fluid found there ordinarily, it is very slight indeed, and lasts for a very short time only. But should pus, or the ichor of decomposition be mingled with it, we should be prepared to expect serious, if not, fatal results.

I once had an opportunity of observing the progress of a case, for several months, where this rupture and effusion were frequently repeated. About every three weeks the woman would attain to a large size, and a well-defined, large cyst could be felt filling up the whole abdomen, and distending greatly ; when suddenly, without premonition or apparent cause, the cyst would give way ; the swelling would become more diffuse ; fluctuation very evident, and the cyst could be no longer defined by the touch ; slight fever and some tenderness of the abdomen would last for two or three days, when copious perspiration and diuresis would evacuate the fluid in a few days more. After this process was completed, the abdomen would be lank, and a small cyst could be felt rising up from the left ilium, it would increase and burst at the end of three weeks, as the other had done before. I saw the patient frequently while this process was repeated six or seven times, when, as she would not submit to the operative procedure, which I insisted upon, I was dismissed, and an irregular practitioner, who was sure he could cure her, installed in my place. Not long, (perhaps three months) after I was discharged, she died from the inflammation resulting from one of these effusions, probably, because the contents of the cyst had become vitiated by inflammation within its cavity.

But these growths may produce a pathological condition of the system, without becoming, themselves, the seat of disease, by the great size they may attain, mechanically interfering with the functions of the pelvic and abdominal viscera. Before rising out of the pelvis, it may displace the uterus and cause inconvenience from this effect; it may, press upon, and obstruct the rectum, bladder, and urethra, or upon the iliac veins, causing obstruction to the flow of blood, and varicose veins in the legs, phlebitis or phlegmasia dolens; or, pressing upon the nerves, cause neuralgic pains in the limbs, hips, etc. It is plain that such pathological effects, when induced, would be serious, in accordance with the greater or less impaction in the pelvis, by its continued growth. Ordinarily, these inconveniences do not prove very embarrassing to the functions of the important vital organs; but sometimes the case is far otherwise, and life is very much shortened, and health rendered miserable. As it rises into the abdomen, these mechanical troubles are apt to be lessened, and as the room is comparatively so great in that cavity, quite a while elapses before any great disturbance results from mechanical pressure; after awhile, however, the abdominal muscles are distended beyond convenient size, and the tumor is strongly pressed among the viscera. The kidneys, liver, stomach, intestinal tube, in fact, all the abdominal organs may become the subject of great, and even fatal pressure. In many instances, however, enormous size is attained before fatal damage results. One hundred and fifty pints of fluid have been taken at a single tapping. A much less amount in most cases, would produce very grave results by pressure. When the growth is rapid, its mechanical effects will be more distressing, and on the contrary, the organs accommodate themselves to a great deal more pressure, if gradually brought about.

Besides the inflammatory changes that take place in the tumor, chronic degeneration is occasionally observed. Deposits of earthy substances in the walls, bony spiculæ, etc., are the most frequent. Small tumors, containing solid material, are more commonly thus affected.

The modes of termination are worthy of some consideration. Many cases last through a great many years without materially influencing the general health, and up to the death of the patient, at an advanced age, prove to be nothing more than an inconvenient burden when large, and when small not the cause of even this kind of trouble. But cases of this class are not very numerous, and by a large majority they terminate and leave the patient in the enjoyment of real or comparatively good health, or by their effect upon the constitution shorten her existence. Spontaneously favorable terminations are so rare that we can base no calculation upon them, but were it possible it would be interesting to follow nature through her resources in this respect; and I am sorry that my means for reference are so limited as to prevent me from thoroughly examining this branch of the subject. Perhaps rupture of the sac into the peritoneal cavity, collapse and adhesion of its walls is the most common and favorable spontaneous termination. After the rupture, in cases where cure follows, it is probable that the opening in the sac continues, and that a permanent fistula, so to speak, from the cyst into the peritoneum, places the fluid in contact with a more active absorbing surface, until by the elasticity of its walls it contracts to annihilation, or at the first shock of the rupture, inflammation is originated that causes an obliteration of the cavity of the sac. Dr. Simpson speaks of instances of evacuation through the vagina. The same thing might occur in connection with the bladder or alimentary canal. I have already spoken of adhesion to, and rupture through the walls of the abdomen, and consequent recovery. Inflammation in its proper tissues, no doubt, sometimes arrests the development of and obliterates the tumor without materially affecting the patient's general health. It is not improbable that other causes, with which we are not acquainted, may likewise operate to the arrest and cure of them, inasmuch as they unquestionably do sometimes disappear in an unaccountable manner.

The local pressure interfering with the functions of the bladder and rectum, may induce complicating diseases that lead to death, and consequently cause death before the tumor is very largely developed. Inflammation will spread upon these organs to their more vital connections and relative organs. Or by interfering with excretion from the bowels or bladder produce disease of the blood, and thus gradually undermine the health of the patient.

After the tumor has arrived at, and greatly distended the abdominal cavity, pressure upon the viscera will sometimes produce disastrous terminations. The stomach is crowded into a very small space, and food can be taken but sparingly, and is often rejected before digestion is completed. The vascular supply of this organ is cramped, and its secretions vitiated and embarrassed, and in this way digestion is interfered with, the appetite destroyed and loathing of food takes its place.

Pressure upon the vena porta embarrasses the secretion of the liver; pressure upon the ductus choledochus, gall-bladder and duodenum, stops the excretion of bile, it is dammed back upon the gland, absorbed and thrown into the blood to poison the nervous centres.

There is no doubt also that the general compression of the organs, by pressure upon the chyle absorbents, prevents that fluid from passing as freely as usual into the blood, and thus by degrees starves the patient. But probably no more disastrous effects of the pressure of the tumor in the abdomen is noticed, than such as is produced through the kidneys. Pressure upon the emulgent veins causes congestion of the kidney tissues, retention of urea, and other matters that should be excreted, and drains off the albumen with the urine, until the blood becomes thinned enough to infiltrate into the cellular tissue generally, in the form of œdema of the extremities, or into the peritoneal cavity, giving rise to ascites. But this is not the worst mischief, perhaps, caused by the pressure on the kidneys; the poisoning of the blood with urea, and its effect on the nerves and vital organs is too well known to require more than mere mention to suggest the rapidly fatal tendencies which result from it.

Inflammation in any of the important abdominal organs, may be caused by the pressure which will terminate fatally in a greater or less time, owing to its acuteness or slowness of progress. It will be seen by the above description, very short to be sure, that ovarian disease usually terminates by inducing a long train of distressing constitutional symptoms. They are not uniform in different cases, some persons suffering from one mode of complication, and some from another, but nearly all are pretty sure to experience those terrible sufferings, connected with secondary disturbances in the vital organs.

